# Physical activity assessment in practice: a mixed methods study of GPPAQ use in primary care

**DOI:** 10.1186/1471-2296-15-11

**Published:** 2014-01-15

**Authors:** Neil Heron, Mark A Tully, Michelle C McKinley, Margaret E Cupples

**Affiliations:** 1 Department of General Practice and Primary Care, Queen’s University, Belfast, Northern Ireland; 2 Centre for Public Health Research, Queen’s University, Belfast, Northern Ireland; 3 UKCRC Centre of Excellence for Public Health Research (NI), Belfast, Northern Ireland

**Keywords:** GPPAQ (General Practice Physical Activity Questionnaire), General/family practice, Physical activity, Physical activity promotion

## Abstract

**Background:**

Insufficient physical activity (PA) levels which increase the risk of chronic disease are reported by almost two-thirds of the population. More evidence is needed about how PA promotion can be effectively implemented in general practice (GP), particularly in socio-economically disadvantaged communities. One tool recommended for the assessment of PA in GP and supported by NICE (National Institute for Health and Care Excellence) is The General Practice Physical Activity Questionnaire (GPPAQ) but details of how it may be used and of its acceptability to practitioners and patients are limited. This study aims to examine aspects of GPPAQ administration in non-urgent patient contacts using different primary care electronic recording systems and to explore the views of health professionals regarding its use.

**Methods:**

Four general practices, selected because of their location within socio-economically disadvantaged areas, were invited to administer GPPAQs to patients, aged 35-75 years, attending non-urgent consultations, over two-week periods. They used different methods of administration and different electronic medical record systems (EMIS, Premiere, Vision). Participants’ (general practitioners (GPs), nurses and receptionists) views regarding GPPAQ use were explored via questionnaires and focus groups.

**Results:**

Of 2,154 eligible consultations, 192 (8.9%) completed GPPAQs; of these 83 (43%) were categorised as inactive. All practices were located within areas ranked as being in the tertile of greatest socio-economic deprivation in Northern Ireland. GPs/nurses in two practices invited completion of the GPPAQ, receptionists did so in two. One practice used an electronic template; three used paper copies of the questionnaires.

End-of-study questionnaires, completed by 11 GPs, 3 nurses and 2 receptionists and two focus groups, with GPs (n = 8) and nurses (n = 4) indicated that practitioners considered the GPPAQ easy to use but not in every consultation. Its use extended consultation time, particularly for patients with complex problems who could potentially benefit from PA promotion.

**Conclusions:**

GPs and nurses reported that the GPPAQ itself was an easy tool with which to assess PA levels in general practice and feasible to use in a range of electronic record systems but integration within routine practice is constrained by time and complex consultations. Further exploration of ways to facilitate PA promotion into practice is needed.

## Background

Non-communicable diseases place an increasingly large burden on healthcare systems worldwide. The World Health Organisation (WHO) has estimated that the major non-communicable diseases accounted for approximately 60% of all deaths annually [1]. A major risk factor for non-communicable diseases is physical inactivity: it has been estimated that physical inactivity accounts for 6-10% of all global deaths annually [2] and is also a major risk factor for disability within our society [3]. Indeed the benefits of physical activity have been well-established by previous authors [4-6], with clear evidence that physical inactivity is a risk factor for coronary heart disease and stroke, osteoporosis, and breast and colon cancer [4]. Thus the prevention of non-communicable diseases and promotion of physical activity is a major priority for healthcare systems and for those who work within them, including those who work within general practice. Physical activity promotion is considered to be a “clinical need, not just a lifestyle choice” [7]. However, whilst effective physical activity interventions have been reported, including pedometer based programmes and exercise referral schemes, levels of promotion and uptake remain low: there has been relatively little investment in researching effective ways to increase physical activity promotion in general practice [8].

The WHO has advised that routine contacts with health-service staff should include practical advice on the benefits of increased levels of physical activity, combined with support for individuals to help initiate and maintain healthy behaviours [9]. Routine and opportunistic physical activity promotion by health professionals has also been endorsed by the UK government [10] and NICE (National Institute for Health and Care Excellence) [11,12]. It is estimated that 78% of people consult their general practitioner (GP) at least once during each year [13,14] and patients view GPs as a reliable source of advice [15,16]. Thus primary care encounters present a major opportunity to prevent disease and to promote healthy lifestyles. This opportunity is of particular relevance for people who are socio-economically disadvantaged. Those in society who are least affluent are also those who have the highest prevalence of chronic disease, requiring ongoing management within the community, and who tend to be least physically active: socio-economic gradients which exist for physical activity now mirror those for health [17]. Linked to physical activity, obesity is also more prevalent in the lower social classes [18].

GPPAQ (General Practice Physical Activity Questionnaire) is a reliable and validated tool for use within primary care to assess adult physical activity levels via seven questions [19] and its use is supported by NICE [12]. Indeed NICE has recently highlighted the need for further research into the use of the GPPAQ in primary care, including the examination of primary care practitioners’ views regarding the questionnaire [12].

Completion of the GPPAQ is estimated to take approximately sixty seconds [8] and generates a simple, four-level physical activity index, categorising patients as: active, moderately active, moderately inactive or inactive. After its initial development [20], GPPAQ was piloted by practice nurses with sixty-one newly registered patients from three practices [21]. Further pilot work was reported from practices in Coventry and London but details of practical aspects of its administration in routine practice and of its use within electronic recording systems other than EMIS (Egton Medical Information Systems Ltd) are limited [21-23]. There are a small number of different information technology (IT) systems used in primary care to record patients’ details: knowledge of GPPAQ integration within these systems is important both for accurate recording of clinical information in relation to providing optimal care for patients as well as for recording data for remuneration purposes.

Whilst the GPPAQ has been used in a UK public health initiative, the ‘Let’s Get Moving’ campaign [7], little is known of its acceptability or of its most appropriate method of administration in routine GP consultations. Indeed, NICE [12] has recently identified a need for further research into the views of health professionals regarding GPPAQ. This study aims to examine methods of GPPAQ administration in non-urgent patient contacts and in different primary care computer systems and to explore the views of health professionals regarding its use within a socio-economically disadvantaged population.

## Method

This feasibility study was conducted using a cross-sectional design in four general practices in Belfast, Northern Ireland, which were specifically chosen because of their position within socio-economically disadvantaged areas, defined by a Multiple Deprivation Measure (MDM) which is derived from their postcode [24] and is an official measure of relative socio-economic disadvantage within Northern Ireland, based on a range of indicators of deprivation. There are 890 MDM rank scores for postcodes in Northern Ireland, 1 being the most deprived and 890 the least deprived. This measure indicated that three practices were located within super-output areas which were ranked in the top 10% of greatest socio-economic deprivation in Northern Ireland (NI), with the fourth in the top 30%. The MDM was also calculated for the individual patients and the majority of participants lived in some of the most deprived areas of Northern Ireland, with 50% of participants within the lowest quintile of scores.

Ethics approval was granted through the Office for Research Ethics Committees NI, reference number 11/NIR03/2, 15/03/2011. The four general practices, all involved in general practitioner (GP) training and undergraduate medical education, used different primary care electronic recording systems and were located in different areas of the city. All agreed to participate and, following discussion of options, each decided their preferred method of administration of GPPAQ to assess physical activity levels for patients aged 35 to 75 years and attending for non-urgent, face-to-face consultation with the GP or practice nurse during a two-week period. Non-urgent consultations were considered to be defined as all appointments other than emergency appointments which were offered to patients who needed to be seen on the day of request; they therefore included patients attending the practices for various reasons, for example blood tests, medication review, symptom management and chronic disease monitoring. Numbers of completed GPPAQ assessments were recorded during two different weeks in each practice between April 2011 and August 2011. Overall, 19 GPs and 10 nurses administered GPPAQs.

Prior to commencement of the study, practices were offered different possible ways of administering the GPPAQ and each identified their preference, in order to minimize disruption to their usual management of the process of patients’ consultations. Options included: (1) the GP or nurse completing the questionnaire directly within the electronic medical record during the consultation; (2) the receptionist providing a paper copy at reception for self-completion by all surgery attendees within the eligible age range for the study; (3) GP/nurse completion of paper-copy during consultation or GP/nurse review of patient-completed paper-copy during consultation, with (i) update of electronic record during consultation or (ii) update of electronic record at a later time. Potentially all receptionists employed in the practices were involved in giving the questionnaires to patients attending but no record regarding who actually did so was kept. Questionnaires were given out during every surgery session during the relevant weeks of data collection.

At the end of the study, health professionals from the four practices (19 GPs and 10 nurses) and two receptionists (one from each practice which used receptionist lead GPAQQ administration), were asked to complete a questionnaire (Additional file 1) to assess the acceptability of the GPPAQ to everyday general practice, using a five-point Likert scale [25,26]. The questionnaire was developed by the study authors for this research and has not been validated. All questionnaires were self-completed by participants without input from the research team.

Two focus groups with the health professionals involved were undertaken at the end of the study and all health professionals from Practices 1 and 2, which each used different methods of GPPAQ administration, were given verbal invites to attend. These included 8 GPs and 4 nurses, with no receptionist involvement. Focus group discussions were digitally recorded with participants’ consent and transcribed verbatim. Two researchers then conducted thematic analysis independently and followed the stages of: familiarisation; identifying a thematic framework; indexing; charting; and, mapping and interpretation [27]. The researchers’ independent findings were discussed by the research team to ensure clear definition of themes and that appropriate supporting evidence was identified for each.

## Results

A total of 2,154 consultations took place with doctors and nurses in the four practices during the study (Table 1). It is not known how many consultations were undertaken by nurses relative to doctors of the total of 2,154 consultations. The practices used different primary care electronic recording systems: two used EMIS, one Premiere GP software and a fourth used Vision. Of note, Practice three, using Premiere software, could not differentiate between telephone and face-to-face consultations, which may have artificially inflated their consultation rate. This research aimed only to include face-to-face consultations and telephone consultations were removed from the other three practices’ consultation figures. Practice four recorded the lowest number of consultations.

**Table 1:** Profiles of participating general practices

**Practice**	**Recruitment periods (2 weeks for each practice)**	**Number of patients per practice (practice size)**	**Number of full-time GP partners in practice**	**Number of nurses in practice**
1	04/04/11 to 17/04/11	8140	5	3
2	18/04/11 to 22/04/11; 28/06/11 to 04/07/11	8829	5	3
3	04/07/11 to 10/07/11; 18/07/11 to 24/07/11	8600	5.5	3
4	25/07/11 to 07/08/11	5009	3	1

Receptionist-led GPPAQ administration, where all eligible patients attending the practice for a face-to-face consultation were given a questionnaire by the receptionist just before their appointment, appears to lead to a greater frequency of questionnaires being completed (Table 2). When the administration was undertaken directly by the health professional, the frequency of completion was less.

**Table 2:** Methods of GPPAQ administration, numbers of eligible consultations and rates of completion in different practices

**Practice**	**Method of GPPAQ administration**	**GPPAQ format**	**Number of GP & nurse consultations for 35-75yo during recruitment period (% of practice size)**	**Number of GPPAQs completed (% of eligible consultations)**
1	GP/nurse-led	Electronic	482 (5.92%)	40 (8.29%)
2	Receptionist-led	Paper-copy	657 (7.44%)	86 (13.09%)
3	GP/nurse-led	Paper-copy	818 (9.51%)	13 (1.59%)
4	Receptionist-led	Paper-copy	197 (3.93%)	53 (26.90%)

Table 3 shows that the majority of GPPAQs in each practice were completed by GPs: overall, nearly 80% of the GPPAQs were completed by GPs. No information was available about the relative numbers of consultations undertaken by nurses during the study compared to doctors. There were 2,154 eligible consultations in the four practices over eight weeks of recruitment: 192 (8.9%) had completed questionnaires, of which 83 (43%) were ‘inactive’ (Table 4).

**Table 3:** Numbers of GPPAQs completed in each practice, with numbers (percentages) completed by GP or nurse

**Practice**	**Total number of GPPAQs completed**	**Number of GPPAQs completed by GPs (% of total for practice)**	**Number of GGPAQs completed by nurses (% of total for practice)**
1	40	38 (95%)	2 (5%)
2	86	60 (69.8%)	26 (30.2%)
3	13	10 (76.9%)	3 (23.1%)
4	53	44 (83.0%)	9 (17.0%)
Total	192	152 (79.2%)	40 (20.8%)

**Table 4:** Numbers and percentages of patients in different categories of activity, as assessed by GPPAQ, within each practice

**GPPAQ category**	**Practice 1 N (%)**	**Practice 2 N (%)**	**Practice 3 N (%)**	**Practice 4 N (%)**
Inactive	29 (72.5%)	36 (41.9%)	6 (46.2%)	12 (22.6%)
Moderately inactive	2 (5.0%)	7 (8.1%)	3 (23.1%)	6 (11.3%)
Moderately active	2 (5.0%)	15 (17.4%)	3 (23.1%)	20 (37.7%)
Active	7 (17.5%)	28 (32.6%)	1 (7.7%)	15 (28.3%)
Total	40	86	13	53

End-of-study questionnaires were completed by eleven of the nineteen GPs and three of the ten nurses, which is a 50% response rate approximately: not all of those who were involved in using the GPPAQ were available at the time of administration of this questionnaire. In addition, the end-of-study questionnaire was completed by the two approached receptionists (Table 5). The health professionals, including the receptionists, generally considered the GPPAQ relatively simple to use, a valuable use of time, and that it could be easily incorporated into their usual consultations (Table 6).

**Table 5:** Numbers of health-professionals’ (N = 16) responses for each point of Likert scale (1 corresponds to ‘strongly agree’ and 5 to ‘strongly disagree’) for each question in end-of-study questionnaires

**Question**	**Likert scale – number of responses**					
	**1**	**2**	**3**	**4**	**5**	0*
1) → I found using the GPPAQ questionnaire straight forward	8	8	0	0	0	0
2) → I found using the GPPAQ a valuable use of time	2	11	2	1	0	0
3) → Using the GPPAQ questionnaire could be easily incorporated into my consultation	1	10	2	1	0	2

*Not applicable (refers to receptionist response).

**Table 6:** **Health professional end-of-study questionnaire responses – 1**^**st **^**quartile, median, 3**^**rd **^**quartile**

	Question 1*	Question 2*	Question 3*
1^st^ quartile	1	2	2
Median	1.5	2	2
3^rd^ quartile	2	2	2

*n = 16 *(11 GPs; 3 nurses; 2 receptionists).

Analysis of focus group data identified two main themes, relating to aspects of GPPAQ administration and the perceived usefulness of GPPAQ questions.

– A) GPPAQ administration.

– 1) Questionnaire structure.

The GPPAQ, a one page hand-out, was considered to be acceptable for use in general practice by all focus group participants. The following comment illustrates clearly how its use was perceived:

“Well it is pretty straight forward to use, I mean it’s self explanatory and each heading gives you a guideline……… I thought it was easy enough”. (Nurse 1, Practice 2).

– 1) Time requirement.

Most health professionals considered that they could incorporate the GPPAQ into their normal consultations and that it could be completed quickly. However, comments revealed how the questions could lead to longer discussions with patients, covering issues that were not directly relevant to the reason for the consultation. Discussions indicated that practitioners perceived that using GPPAQ could extend the time required for consultations. That is, the GPPAQ itself did not take long to complete but the resulting discussion might have required a longer consultation which was not always possible. The interviewers’ observations of non-verbal communications during the focus group confirmed unanimous agreement amongst the health professionals that this was a potential impact of its use.

“……….. it didn’t take too long to complete”. (Doctor 4, Practice 1).

“ ……….keeping them focused on what exactly you were asking them and not getting them to expand on it too much”. (Nurse 2, Practice 2).

Time pressure was the main issue cited by the health professionals for not completing the GPPAQ. Some participants reported how they had planned to manage this proactively, pre-empting questions, arranging personal reviews, and sometimes referring to another member of the primary care team. Several participants reported how a 10-minute consultation was already too short to deal with some patients’ multiple problems, however, they also recognised that these patients may be denied the potential for health benefits if not given appropriate advice about physical activity.

“the consultations are so pressurised……….and the thing is, the type of patients which could have benefited from this, were those patients with maybe multiple pathologies – complex patients”. (Doctor 2, Practice 1).

– 1) Questionnaire format.

Overall it appeared that most health professionals preferred the GPPAQ paper-copy method of administration, with self-completion by the patient prior to entering the consultation. Having a paper copy presented to them meant that it was less easy to forget to activate the electronic record during consultations. In Practice 1, following a suggestion made by one of the GP partners, a paper copy was taped to each desk as a reminder for its use. Such prompts were viewed as useful in addressing the issue of physical activity within consultations, particularly in consultations where this aspect of health promotion may not have been viewed as a priority.

“I liked …… a reminder because in so many consultations I wouldn’t have broached the issue for the reasons that you say about complex consultations”. (Doctor 3, Practice 1).

“……so the patient is given a questionnaire and they come in with it filled out and it’s a way of opening a conversation about it”. (Doctor 2, Practice 2).

In contrast however, one GP commented on the ease of use of the questionnaire being integrated within the electronic recording systems although EMIS was the only primary care computer system which allowed full integration of the GPPAQ within the electronic clinical record. That doctor also highlighted the usefulness of electronic recording of information that could be used in a future consultation.

“……..you could link it in and save it to the consultation…… even if you never had time to talk about it a lot on that day, when they were back in you could bring it up in discussion”. (Doctor 1, Practice 1).

The information that most GPPAQs were completed by doctors rather than nurses was presented to each focus group: no-one suggested that it was an inaccurate reflection of practice. However, doctors’ comments revealed how they had perceived that nurses would have more time for such discussions and would have used GPPAQs more readily. None of the nurse participants offered possible explanations.

“That is strange cause nursing staff commonly project the idea that they have a much more conversational type of relationship with patients, right, and this (GPPAQ) would have naturally lent itself to that type of style”. (Doctor 2, Practice 1)

– B) Usefulness of questionnaire information.

The health professionals considered that the GPPAQ output was easy to understand and the resulting assessment of physical activity level, described in simple categories, was easily conveyed to patients. It was perceived that its use facilitated discussion of issues relevant to health promotion, particularly in consultations in which practitioners were aware of multiple or complex problems and time constraints.

“…………to actually have an objective measure of the activity level so you could say to the patient, you know this questionnaire says you’re not active”. (Doctor 3, Practice 1).

“....it lent itself to trying to do more health promotion that you wouldn’t have been able to do or wouldn’t have done otherwise in a pressurised consultation”. (Doctor 1, Practice 1).

A current lack of awareness of how physical activity assessment relates to practice funding was illustrated by a request for clarification about current policies.

“Is there any money for recording physical activity levels or promoting it?”. (Doctor 2, Practice 2).

## Discussion

These findings show that, despite primary care health professionals reporting that the GPPAQ can be used easily within the setting of everyday general practice amongst a socio-economically disadvantaged population, it was used in a minority of consultations. Less than 10% of consultees attending over the 8 weeks of study had a GPPAQ performed. The complexity of patients’ problems add to the time required for meaningful discussion of responses to the questionnaire: constraints on its use are mainly related to time. Overall, the most efficient method of GPPAQ administration appeared to be to provide a paper copy of the questionnaire to all patients, for completion in consultation with the health professional.

### GPPAQ and study participants

Our finding that the GPPAQ can be used within routine general practice concurs with previous reports from a convenience sample of six London GP practices with a large Asian or Asian British population [22] and two other small studies [21]. However, no information was given regarding the socio-economic status of previous study participants or of the use of GPPAQ with electronic systems other than EMIS. Thus our study provides new information regarding its suitability for use with patients from socio-economically disadvantaged areas, within routine consultations in everyday practice and in conjunction with additional electronic record systems. This information is relevant to those who live and practice in such areas, in order to promote physical activity and to attempt to reduce recognized health inequalities [17].

In comparing different ways of administering the GPPAQ our study also provides novel information. Receptionists providing paper-copies to patients appeared to evoke a relatively higher rate of completion compared to the health professionals administering the GPPAQs, either using paper-copies or electronic formats, because the visual stimulus of the patient walking into the consultation with the questionnaire appeared to trigger the health professional to complete it. Electronic records have clear advantages over paper records in that they provide an easily accessible record of assessment and a resource for reference in future consultations. The ease of transfer of information to an electronic record varies between different systems, with the EMIS computer system appearing the most compatible with the electronic version of GPPAQ as it allows the GPPAQ score to be directly integrated into the EMIS patient notes. Further work is required on integrating GPPAQ within all the electronic recording systems used within primary care.

It is of interest that, of the 2,154 consultations during the study period, only 192 (8.9%) had a GPPAQ completed. This rate is similar to the 6% reported from London practices [23]. Time constraints were cited as the main reason for low rates of completion. Also, some health professionals considered that physical activity assessment was not appropriate in every consultation. Our focus group data indicated that health professionals gave consideration both to the time required to discuss the outcome of the GPPAQ with their patient and to the potential value of that discussion for the patient, which is in keeping with previous reports that health professionals made subjective appraisals of their patients’ suitability prior to completion of a GPPAQ and recruitment to a physical activity intervention [23]. In some instances it may be suggested that they tended to select patients whom they deemed ‘inactive’ for invitation to participate. Findings from Practice 1, in which health professionals administered the GPPAQs and 72.5% were classed as ‘inactive’ may support this suggestion – this rate of inactivity is higher than expected, even in an area of socio-economic deprivation, in comparison to a reported prevalence of approximately 40% in the general population [28]. In Practice 4 receptionists gave the GPPAQ to patients who then had greater opportunity to influence decisions regarding its completion: in these circumstances the rate of completion of the GPPAQ was higher and the proportion of inactive patients was lower, suggesting that a health professional’s perception of a patient’s level of inactivity made a lesser contribution to GPPAQ completion. However, further work would be required to fully explain the variations in completion rates and levels of physical inactivity found in different practices.

The evaluation questions completed by health professionals showed that they considered the GPPAQ straight-forward to use, a valuable use of their time and that it could be incorporated into their day-to-day consultations, which was consistent with the focus group findings. This concurs with reports of previous studies [22,23] and helps to address NICE’s recent call for further research into primary care practitioners’ views of GPPAQ [12]. However, as highlighted in this study, there still appears to be several barriers to discussing physical activity within most consultations in UK general practice.

Factors often cited for a reluctance to discuss physical activity promotion are: time (ten minute consultations) [29]; a feeling of ineffectiveness by the practitioner; inadequate training; lack of financial reimbursement; relevance to the patient’s presenting compliant; perceptions of poor patient compliance; lack of appropriate tools to assess and prescribe exercise; and, impact on the clinician-patient relationship [14-16,30,31]. These findings were supported by comments in our focus groups.

Moreover health professionals who are themselves physically active are more likely to promote physical activity [32]. This study suggests that one method of promoting discussion in general practice about physical activity is to offer patients a GPPAQ to complete, particularly via the receptionist with a paper-copy, and in order to facilitate this discussion, to provide physical activity education to GP trainees as well as in the undergraduate medical curriculum [33,34]. Promoting knowledge and interest in physical activity among health professionals is important [35]. Whilst we were aware that at least one GP in each participating practice had a keen personal interest in being physically active, we did not formally assess the health professionals’ levels of activity. It is possible that levels of completion of GPPAQ in other practices with dis-interested health professionals may have been less.

With regard to time pressures and completion of GPPAQ reported here and elsewhere [29], this study approached GPPAQ completion with minimal intrusion on consultation time, included self-completion by patients prior to face-to-face consultation with a health professional and subsequent integration of the assessment into an electronic record system. All of the electronic computer systems included in our study are capable of accommodating this approach.

Recent papers have shown that including a health parameter within the Quality and Outcomes Framework in British general practice, generally leads to an improvement in care [36]. Physical activity is currently minimally rewarded within the Quality and Outcomes Framework and only within certain parts of the UK (United Kingdom) [37]. Greater recognition within this reward system may encourage physical activity assessment and promotion and help translate the policy of physical activity promotion into practice: it would be in keeping with the concept that physical activity promotion provides the ‘best buy in public health’ [38].

We were unable to determine the number of consultations completed by nurses compared to doctors in each practice. The finding that 79.9% of GPPAQ questionnaires were completed by doctors is not in keeping with general expectations that nurses would have completed the majority, given their role in the provision of routine health promotion advice [15]. Nurses are thought to be in an ideal situation to discuss physical activity promotion through, for example, patients attending for blood pressure measurement or for monitoring of cholesterol levels or diabetes. Their perceptions of the use of GPPAQ were similar to those of the GPs, recognising that the tool itself was easy to use but its use could present challenges in time management. However the study only included four practices and the doctors may have taken ownership of questionnaire administration which may not necessarily be reflected in their attitude in usual day-to-day working practice.

### Strength and weaknesses

The study involved a small number of general practices but it was designed as a feasibility study: its strength is that it has examined GPPAQ use in a population different from those reported previously and it has provided details of different methods of administration. The practices were selected from socio-economically disadvantaged areas, the Multiple Deprivation Measure scores confirmed their level of disadvantage and it is in such a population that there is greatest need to promote lifestyle change and increase levels of physical activity. The study has included three different electronic record systems and whilst it is difficult to compare the efficiency of these different systems in using GPPAQ, we have identified that it is feasible to work with them. Our findings will inform the design of future study of the assessment and promotion of physical activity in primary care.

It is difficult to compare rates of GPPAQ completion between practices, since observations took place over different time periods. However, both nurses and doctors contributed to the study, indicating the acceptability of the tool to different professionals. We omitted to include receptionists in our focus groups – for future work, arrangements would be made to ensure that reports of their experiences of administering the questionnaires would be explored. The patients who never entered the study did not consent for the researchers to access their data: no details are therefore available about them and this may well have provided important information about the background population. We do not know whether every potentially eligible patient was invited or if health professionals used other criteria to select those for invitation to compete the questionnaire. Completion of GPPAQ in only 8% of consultations was a low rate of implementation and the lack of information about those who declined or who were not invited to complete a GPPAQ limits the generalisability of our findings to routine practice.

The response on end-of-study questionnaires was rather low (50%) by GPs and nurses. This was due to asking the health professionals to complete the questionnaire within their everyday workload and during a limited time interval during which some who had participated in the study were absent from their practice. However, we don’t believe this is a limitation of our study as the end-of-study results were discussed and confirmed in the focus group.

## Conclusions

The current emphasis, particularly within the UK following the 2012 Olympics, on the value for well-being from participating in physical activity requires positive efforts to translate policy into practice. Our findings show that it is feasible using GPPAQ as a brief assessment tool to discuss physical activity in general practice consultations. It is viewed as being easy to use and it provides an objective measure of physical activity which can be conveyed to patients and forms a basis for advice. Our study identifies how nurses and doctors in primary care identify time constraints related to dealing with patients’ presenting problems which limit their capacity to initiate discussions about wider issues relevant to health promotion. Whilst this may be addressed by giving greater recognition to the importance of teaching health professionals about effective methods of health promotion, greater recognition of the value of these efforts might also be given within the Quality and Outcomes Framework, rewarding performance in this area of clinical care. Such initiatives may result in higher levels of participation in physical activity by the population but more research is also required to determine the most effective methods of supporting implementation of the policy of physical activity promotion by primary care practitioners.

## Competing interests

The authors declare that they have no competing interests.

## Author’s contributions

NH, MAT and MEC conceived the study. NH carried out the study, performed the statistical analysis, and drafted the manuscript. All authors participated in the design of the study, with MAT, MCM and MEC providing supervision throughout the study, helping with study analysis and reviewing successive drafts of the manuscript. All authors read and approved the final manuscript.

## Pre-publication history

The pre-publication history for this paper can be accessed here:

http://www.biomedcentral.com/1471-2296/15/11/prepub

## Supplementary Material

Additional file 1 Health professional study satisfaction questionnaire. Click here for file
