# Supplementary material for: Physical activity assessment in practice: a mixed methods study of GPPAQ use in primary care
Source: BMC Fam Pract. 2014 Jan 15;15:11. doi: 10.1186/1471-2296-15-11 (PMC3897938; doi:10.1186/1471-2296-15-11)
Supplement: Additional file 1 — Health professional study satisfaction questionnaire. [file 1471-2296-15-11-S1.doc]

**Appendix 1**

Health professional study satisfaction questionnaire

This is a survey about your attitudes towards the GPPAQ questionnaire and of a potential pedometer-based physical activity programme.

Professional completing the questionnaire: **doctor/nurse/other** (if other please specify) (please circle the appropriate response)

Please read the following statements and circle the response that MOST CLOSELY represents your view:

**I found using the GPPAQ questionnaire straight forward:**

Strongly agree agree neither agree nor disagree disagree strongly disagree

Comments

**I found using the GPPAQ a valuable use of time:**

Strongly disagree disagree neither agree nor disagree agree strongly agree

Comments

**Using the GPPAQ questionnaire could be easily incorporated into my consultation:**

Strongly agree agree neither agree nor disagree disagree strongly disagree

Comments

If you have any further comments regarding this study, GPPAQ questionnaire and/or a pedometer-based physical activity programme, we would be grateful for these in the box below:
